# Safety of postimplantation MRI with Dixi microdeep electrodes in situ: An in vitro evaluation of MRI‐related heating at 1.5T

**DOI:** 10.1002/epi4.70238

**Published:** 2026-02-20

**Authors:** Ruth O’Gorman Tuura, Roger Luechinger, Rahel Heule, Raimund Kottke, Sabine Rona, Niklaus Krayenbühl, Georgia Ramantani

**Affiliations:** ^1^ Center for MR Research University Children’s Hospital Zurich Switzerland; ^2^ Children’s Research Center University Children’s Hospital Zurich Switzerland; ^3^ Institute for Biomedical Engineering University and ETH Zurich Zurich Switzerland; ^4^ Department of Diagnostic Imaging University Children’s Hospital Zurich Switzerland; ^5^ Swiss Epilepsy Center, Klinik Lengg Zurich Switzerland; ^6^ Division of Pediatric Neurosurgery University Children’s Hospital Zurich Switzerland; ^7^ Department of Pediatric Neurology University Children’s Hospital and University of Zurich Zurich Switzerland

**Keywords:** epilepsy, icEEG, MRI safety, RF heating, SAR, stereoencephalography

## Abstract

**Objective:**

Postimplantation assessment of the position of depth EEG electrodes for intracerebral recordings in patients with refractory focal epilepsy can be performed with MRI or with CT after coregistration to a preimplantation MRI. While both methods offer risks and advantages, postimplantation MRI risks depend on the electrode heating profile under different MRI conditions. We aimed to assess the MRI‐related heating of Dixi microdeep electrodes at 1.5T in multiple electrode configurations and with varying levels of radiofrequency (RF) power.

**Methods:**

In vitro tests of heating due to RF power deposition were performed according to the F2182‐19e2 standard from the ASTM (American Society for Testing and Materials International). A 10‐contact Dixi microdeep electrode was inserted into the gel within the ASTM head–torso phantom, and the temperature was recorded from selected electrode contacts during MRI. Tests were performed with the electrode positioned in various locations in straight and coiled configurations, with coil diameters from 6 to 25 cm. MRI was conducted on a 1.5T Philips Achieva scanner using the transmit–receive body coil.

**Results:**

Significant heating was observed for all configurations where more than 12 cm of the electrode was in the RF coil, apart from those with an applied specific absorption rate (SAR) ≤0.16 W/kg and with additional coiling of the electrode lead using a diameter of ≤6 cm. The worst‐case configurations, reaching a maximum temperature of 70°C (temperature rise 48°C), occurred where the electrode end was straight or looped with a large‐diameter (25 cm) loop. Heating was greatest in the contact furthest from the tip.

**Significance:**

Dixi microdeep electrodes demonstrate heating levels capable of causing serious injury during MRI, but using a conservative SAR limit of 0.1 W/kg and coiling the electrode lead to a diameter of ≤6 cm appears to reduce the heating risk.

**Plain Language Summary:**

Electrodes positioned within the brain for planning epilepsy surgery can heat up during MRI. Using a standard test object mimicking the electrical properties of the human body, we measured heating of Dixi microdeep depth electrodes in different positions and orientations and with varying levels of radiofrequency power. We found substantial heating apart from when the radiofrequency power was greatly restricted or when the lead was tightly coiled. Different electrode contacts showed drastically different heating, and heating levels capable of causing serious injury were measured during MRI.

**Key points**

- Dixi microdeep SEEG depth electrodes can heat significantly during MRI.
- Limiting the specific absorption rate (SAR) to 0.1 W/kg reduces the heating, but safe scanning cannot be guaranteed in all scenarios.
- Heating was greatest at the most proximal electrode contact, furthest from the tip.
- Future tests of RF heating should test multiple contacts and not just the tip contact within SEEG electrodes.

## 1 INTRODUCTION

Epilepsy is a common neurological condition affecting 50–70 million people worldwide,^1^ with a lifetime prevalence of 7.6 per 1000 persons.^2^ On average, 25%–40% of patients diagnosed with epilepsy will develop drug resistance,^3^ leading to recurrent, uncontrolled seizures with a significant loss of quality of life. Refractory focal epilepsy is amenable to surgery in selected cases, but the success of epilepsy surgery depends heavily on the correct identification and full resection or disconnection of the epileptogenic zone (EZ). In patients without a clear lesion on MRI, identification of the EZ typically necessitates a multimodal presurgical evaluation, invariably involving carefully planned invasive EEG recordings targeted to the presumed EZ, together with detailed anatomical verification of electrode location to interpret the recordings and guide surgical planning.

Stereoelectroencephalography (SEEG) using depth electrodes is increasingly used in invasive presurgical evaluation^4^, ^5^ due to its ability to record from deep cortical or subcortical regions (unlike subdural grid and strip electrodes) and the superior spatial resolution of the recordings. SEEG has become the most commonly used invasive EEG method in the USA, according to data from the Centers for Medicare and Medicaid Services.^4^ It offers a higher diagnostic yield compared to subdural electrodes, enabling more precise delineation of the EZ, and is associated with better tolerability and lower risk of serious complications,^3^ as consistently reported in meta‐analyses.^6^ The increasing adoption of robotic systems for electrode implantation^3^ is expected to further drive SEEG use, offering an optimal tradeoff of precision vs. invasiveness.^6^

As with other invasive methods, postimplantation imaging of SEEG electrodes is essential to accurately verify their spatial position relative to the surrounding anatomy, localize the EZ, and guide resection or disconnection planning when indicated. This can be performed either with CT after coregistration to a preimplantation MRI, as originally performed,^7^, ^8^ or with intra‐ or postoperative MRI as increasingly done in recent years.^9^, ^10^, ^11^, ^12^ While CT remains in use, even where MRI is available, due to its lower susceptibility to artifacts and geometric distortion,^13^ MRI offers superior soft tissue contrast and avoids ionizing radiation, making it a viable option when safety conditions are met. For sites considering performing postimplantation MRI with electrodes in situ, the safety profile of the electrode under different scan conditions is critically important. In addition, site‐specific factors such as the availability of intra‐ or postoperative MRI, local MR safety expertise, and the availability of an established CT/MR image registration and fusion pipeline^14^ may influence the choice of imaging modality.^15^

While CT is associated with exposure to ionizing radiation, performing MRI with electrodes in situ carries a risk of heating, which can be significant and can lead to serious injury.^16^, ^17^, ^18^ Radiofrequency (RF) burns are the most commonly reported injury occurring during MRI examinations,^19^ and heating of conductive materials can arise from local currents generated by the changing electric field or from magnetic induction. RF heating can increase dramatically if a resonance condition is reached, e.g., through the formation of a resonant antenna^20^ or a resonant loop.^21^ In addition, recent studies have demonstrated significant coupling between wires in multiwire conducting implants, such that the heating of each wire depends not only on its own geometry but also depends on the locations and lengths of neighboring wires.^22^ This interwire coupling effect was observed for partially immersed wires, a configuration highly relevant in invasive EEG recordings. However, only one previous study investigated the heating profile of Dixi microdeep SEEG electrodes,^23^ and several questions remain. These include the extent of heating across different electrode contacts, the impact of electrode configuration, and the effects of varying RF power transmitted during scanning. Unlike deep brain stimulation electrodes, which are implanted into fixed anatomical targets, SEEG electrodes may be placed in a wide range of locations depending on the presumed EZ, leading to substantial variability in implantation geometry. As such, there is no single typical implantation pathway, and implantation schemes can differ substantially between patients, both in terms of the positioning of the electrodes and the number of depth electrodes implanted. This variability makes it challenging to predict the heating for all clinically applicable configurations. However, since part of the electrode lead remains external to the patient, modifying the configuration of the external portion may help reduce heating risk. In addition, testing of worst‐case configurations can help define conservative safety limits for MRI use. Therefore, the purpose of the present study was to assess the MRI‐related heating of the Dixi microdeep electrodes at 1.5 T across a range of configurations including anticipated worst‐case scenarios, and with varying levels of RF power. While these electrodes were previously labeled as MR conditional,^23^ they are currently not labeled for MR safety.

## 2 METHODS

In vitro tests of RF‐induced heating effects were performed according to the F2182‐19e2 standard from the American Society for Testing and Materials International (ASTM), using the ASTM head‐torso phantom, filled with gelled saline (deionized water with 1.32 g/L NaCl and 10 g/L polyacrylic acid). The electrical and thermal properties of the gel are formulated to match those of human tissue, with a conductivity of 0.47 S/m at 64 MHz, comparable to the conductivities of gray and white matter (0.511 S/m and 0.292 S/m, respectively).^24^ Experiments were performed in two different measurement sessions with a Dixi microdeep SEEG electrode with 10 platinum/iridium electrode contacts (2 mm length, 0.8 mm diameter) and a 100 cm lead.

Tests were performed with the externalized lead in a variety of configurations (see Table 1 for details), including a straight configuration along the midline and at fixed offsets in the right–left and superior–inferior directions, as well as configurations expected to represent worst‐case heating. Both fully and partially immersed placements within the RF body coil were tested, with the electrode inserted into the phantom with some of the lead externalized in air. For the straight line configuration, the electrode was positioned straight along the *z* axis, but the lead was angled slightly anteriorly from the surface of the gel to the edge of the phantom, and then posteriorly from the edge of the phantom to the scanner table, where the free end of the lead was secured (Figure S1). The body coil was used for RF transmission since most commercial head coils are receive‐only. Additional tests were performed with the electrode lead coiled into loops ranging from 6 to 25 cm in diameter. For all configurations, the electrode lead was in the open condition, unconnected to the amplifier, since this is the typical scenario when performing postimplantation MRI in the clinical setting.

**TABLE 1:** Electrode configurations and heating.

Configuration (Figure 1)	Electrode location	Externalized lead configuration	wbSAR (W/kg)	Contacts tested	*T*_max_ contact 1	Δ*T* contact 1	*T*_max_ contact	Δ*T* contact
					(°C)	(°C)	10 (°C)	10 (°C)
Session 1								
A	3.5 cm from left edge of phantom (torso section), center of RF coil	Straight, oriented superiorly along the *z* axis	3.3	1, 2, 10	23	2	67	46
A	3.5 cm from left edge of phantom (torso section), center of RF coil	Straight, oriented superiorly along the *z* axis	0.70	1, 2, 10	21	1	28	8
A	3.5 cm from left edge of phantom (torso section), center of RF coil	Straight, oriented superiorly along the *z* axis	0.16	1, 2, 10	21	0	22	2
Session 2								
A	3.5 cm from left edge of phantom (torso section), center of RF coil	Straight, oriented superiorly along the *z* axis	3.3	1, 3, 8, 10	22	1	49	28
B	3.5 cm from left edge of phantom (torso section), 15 cm inferior to center of RF coil	Straight, oriented superiorly along the *z* axis	3.3	1, 3, 8, 10	23	1	>70	>47
C	3.5 cm from left edge of phantom (torso section), 15 cm superior to center of RF coil	Straight, oriented superiorly along the *z* axis	3.3	1, 3, 8, 10	22	0	24	1
D	3.5 cm from left edge of phantom (torso section), center of RF coil	Straight, oriented inferiorly along the *z* axis	3.3	1, 3, 8, 10	24	2	68	46
E	13.5 cm from left edge of phantom (torso section), center of RF coil	Straight, oriented superiorly along the *z* axis	3.3	1, 3, 8, 10	22	1	44	23
F	23.5 cm from left edge of phantom (torso section), center of RF coil	Straight, oriented superiorly along the *z* axis	3.3	1, 3, 8, 10	22	1	49	29
G	Midline, center of RF coil	Looped in coronal plane, diameter 25 cm	3.3	1, 3, 8, 10	23	1	>70	>47
H	Midline, center of RF coil	Looped in coronal plane, diameter 13 cm	3.3	1, 3, 8, 10	23	1	44	16
I	Midline, center of RF coil	Looped in coronal plane, diameter 8.5 cm	3.3	1, 3, 8, 10	23	1	38	14
J	Midline, center of RF coil	Looped in coronal plane, diameter 6 cm	3.3	1, 3, 8, 10	22	0	27	3
J	Midline, center of RF coil	Looped in coronal plane, diameter 6 cm	0.16	1, 3, 8, 10	23	0	25	1
K	3.5 cm from left edge of phantom (torso section), center of RF coil	Looped in coronal plane, diameter 6 cm	3.3	1, 3, 8, 10	23	0	27	4
L	Midline, center of RF coil	Looped in axial plane, diameter 6 cm	3.3	1, 3, 8, 10	22	0	24	0

Temperature measurements were recorded using a Neoptix fluoroptic thermometry system (Device REFLEX, 4‐channel fiber optic conditioner). In session 1, three electrode contacts were monitored, and a fourth probe recorded the background gel temperature. In session 2, temperature was recorded from four electrode contacts within the electrode (see Table 1 for details). Temperature sensors were attached to electrode contacts with string and inserted into the torso section of the ASTM phantom, where the electric field is highest and most homogeneous^25^, ^26^ (see Figure S1 for a close‐up picture of the electrode and an overview of the experimental setup). The electrode was inserted to a length of 10 cm, at a 30 degree angle such that the tip was positioned 5 cm below the surface of the gel (Figure S2). A Styrofoam sheet was used to secure the lead in air above the gel, analogous to the clinical scenario where the electrode is partially immersed while the externalized lead remains in air outside the head. According to ASTM F2182‐19e2, the region of highest electric field is near to the lateral tank wall; this was confirmed by reference measurements and was consistent with findings from previous studies.^26^ During session 1, contacts 1, 2, and 10 were monitored, while in session 2, contacts 1, 3, 8, and 10 were recorded. Contact 1 corresponds to the most distal (tip) contact.

MRI was performed on a 1.5T Philips Achieva scanner using the transmit/receive body coil and a turbo spin echo sequence with a field of view (FOV) of 530 mm, an acquisition matrix of 380 × 256, an echo time (TE) of 9.9 ms, a repetition time (TR) of 2156 ms, 2 slices, a flip angle of 90°, a turbo factor of 128, and 14 dynamics. The system‐reported whole‐body specific absorption rate (wbSAR) was 3.3 W/kg, and the time‐averaged root mean square transmit field (B1 + rms) was 4.53 μT. In each session, additional scans were performed at reduced wBSAR levels of 0.7 (B1 + rms 2.03 μT) or 0.16 W/kg (B1 + rms 1.01 μT), by setting the user‐defined SAR mode accordingly. Each heating measurement lasted up to 3 min; scans were aborted early if the temperature in any of the contacts exceeded 70°C. Between measurements, scanning was paused to allow the gel temperature to return to baseline. In a separate measurement session outside the scanner, the resistivity was tested between all contacts in the electrode tip and all pins in the connector, to verify that each electrode contact was connected to a single insulated lead, terminating in the connector.

## 3 RESULTS

**FIGURE 1:**
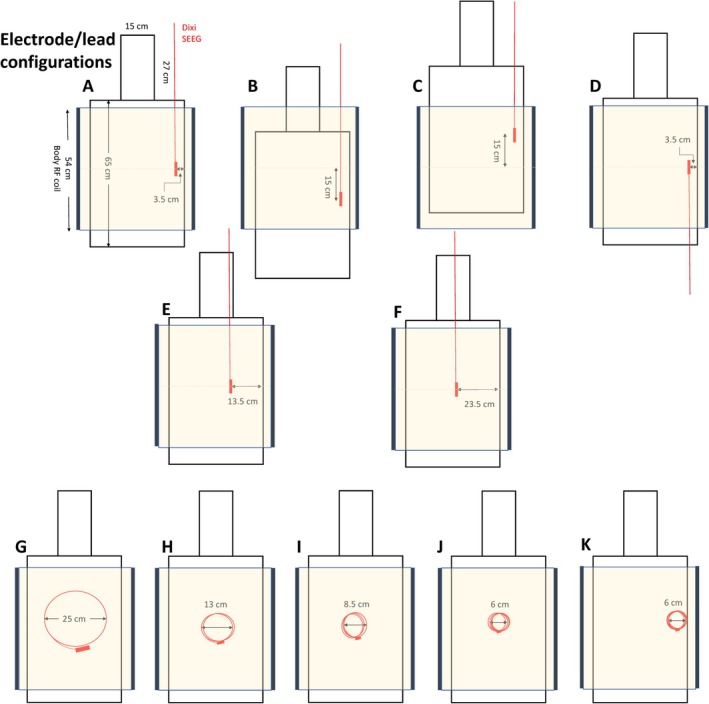
Electrode and lead configurations utilized in the experiments. The depth electrode is depicted in red, within the outline of the ASTM head‐torso phantom, and the volume of the RF body coil is depicted in yellow.

Figure 1 depicts the electrode and lead configurations, and further details regarding the experimental setup and maximum temperature rise for each configuration are given in Table 1. Significant heating was observed for configurations where more than 12 cm of the electrode lead was in the RF coil, apart from those with wbSAR ≤0.16 W/kg and with additional coiling of the electrode lead using a diameter of ≤6 cm. The background temperature rise recorded in the gel during session 1 was 0.5°C during a 3‐min scan with a wbSAR of 3.3 W/kg.

### 3.1 Sessions 1 and 2: Straight‐line orientation

**FIGURE 2:**
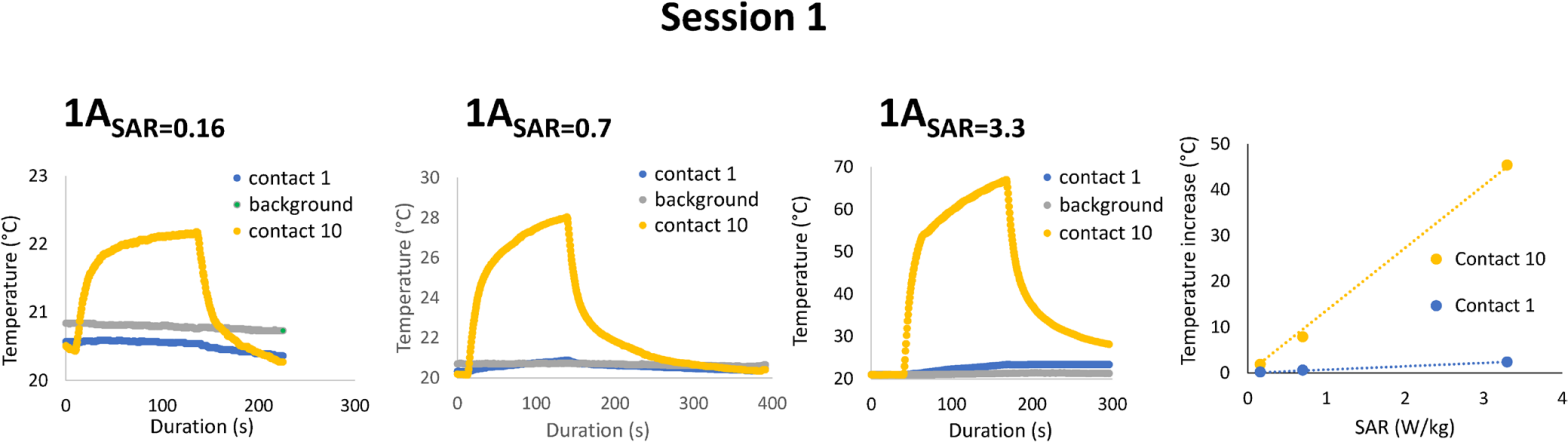
Heating curves from the electrode contacts during each run in session 1, with the electrode/lead configuration depicted in Figure 1A. Heating was measured in the same worst‐case location in the left thoracic region of the tank in all experiments, with wbSAR levels of 0.16, 0.7, and 3.3 W/kg. The heating within electrodes 1 and 10 (the most distal and proximal electrodes) is shown in comparison to the background heating (panels 1–3). The linear relationship between wbSAR and heating is shown in panel 4.

In session 1, the electrode was positioned in a straight‐line orientation with the lead oriented superiorly along the *z* axis, and the tip positioned 3.5 cm from the left edge of the torso section of the phantom. At a wbSAR of 3.3 W/kg, the distal (tip) contact showed a temperature rise of 2°C, whereas the most proximal contact demonstrated a rise of 46°C. When wbSAR was reduced to 0.16 W/kg, the same configuration resulted in a 2°C rise at the most proximal contact (see Figure 2 for heating curves as a function of applied wbSAR). Based on the linear relationship between SAR and heating, a wbSAR limit of <0.1 W/kg would be expected to limit the maximum heating to 1°C in this worst‐case configuration.

**FIGURE 3:**
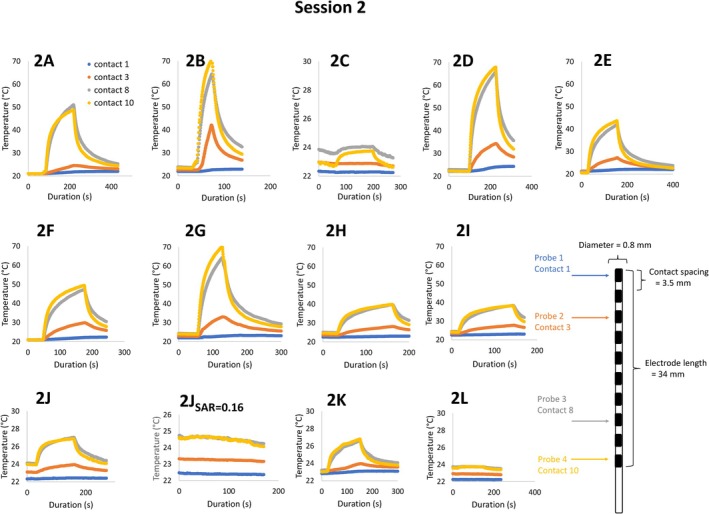
Heating curves from the electrode contacts during each run in session 2, with the electrode/lead configuration depicted in Figure 1A–L. All heating measurements were acquired with wbSAR = 3.3 W/kg unless otherwise specified (e.g., in panel 2J). Heating was measured in contacts 1, 2, 8, and 10, and in all experiments, the highest heating was seen in the most proximal contacts furthest from the tip (contacts 8 and 10).

In session 2, the electrode was repositioned in the left thoracic region of the tank, in the same position and straight‐line orientation as for session 1. Heating measurements were then performed with the electrode in the center of the RF coil, and at table offsets of 15 cm in the inferior and superior directions using a wbSAR of 3.3 W/kg. A maximum heating of 48°C, leading to early termination of the scan as the proximal contact reached a temperature of 70°C, was observed with an inferior offset of 15 cm from the center of the RF coil, where 42 cm of the lead (oriented superiorly to the electrode tip) was within the RF coil (configuration B, Figures 1 and 3). In contrast, heating of only 1°C was observed for a superior offset of 15 cm (configuration C), where only 12 cm of the lead was inside the RF coil (see Figure 1 for electrode configurations, and Figure 3 for corresponding heating curves).

Subsequent experiments tested the heating as a function of the position in the right–left direction, with the electrode at 3.5 and 23.5 cm from the edge of the tank. While highest heating occurred near the left lateral edge of the phantom, consistent with the nonuniform electric field distribution, substantial heating (with a temperature rise of more than 20°C) was also observed with the electrode positioned along the midline of the tank.

The straight‐line configurations aligned along the *z* axis, repeated across both measurement sessions after repositioning of the phantom, electrodes, and temperature probes in the left thoracic region, resulted in similar heating profiles for configurations A and B, in which 27–42 cm of the electrode lead was inside the RF coil. However, the difference in heating measured in configuration A between sessions 1 and 2 (18°C) underscores the experimental uncertainty when measuring near a resonance condition, where small changes in experimental setup can lead to large difference in heating.

### 3.2 Session 2: Looped orientation

In session 2, additional tests were performed with the electrode lead looped in the coronal plane with loop diameter ranging from 6 to 25 cm. Maximum heating was observed with the 25 cm diameter loop, leading to early termination of the scan as the proximal contact reached a temperature of 70°C. Heating was reduced with narrower diameter loops, but even the 6 cm loop produced 3–4°C of heating depending on the loop position. After reducing the wbSAR to 0.16 W/kg the heating was reduced to 1°C. No heating was observed with the lead coiled in a 6 cm loop in the axial plane.

## 4 DISCUSSION

Dixi microdeep® electrodes demonstrate significant heating during MRI at 1.5T, particularly when the electrode is straight or coiled with a loop diameter >6 cm. Interestingly, the highest heating was observed at the most proximal contact furthest from the tip while the lowest heating was observed at the tip contact. This result indicates substantial interwire coupling may be present, although we were not able to assess this coupling directly. Coupling effects have been described previously in other multiwire conductive implants,^22^ although the 14–50‐fold difference in heating between distal and proximal contacts observed here exceeds values previously reported.^22^, ^27^ These findings highlight the need for future RF safety evaluations to assess multiple contacts along SEEG electrodes rather than assuming tip‐only measurements are representative, since each contact corresponds to the end of a separate wire.

In clinical practice, the distal and proximal contacts sample both epileptogenic and healthy brain tissue—within the EZ to help delineate the seizure onset zone, and in surrounding healthy areas to elicit responses and map eloquent functions to be spared during resective or disconnective surgery. While the most proximal contact may lie outside the brain, depending on the electrode length and the depth of the targeted region, it is usually positioned within brain tissue for recording. Depending on its placement, heating of this contact may cause thermal damage to epileptogenic areas or even to healthy cortex.

For grid electrodes, previous studies have reported less RF heating with an increasing number of contacts.^28^ However, the coupling effects are likely to differ between subdural and depth electrodes due to their differing geometries, so it remains unclear whether the same pattern applies to SEEG electrodes. Previous work has also investigated combinations of subdural and depth electrodes, reporting a maximum temperature increase of 2.5°C for Ad‐Tech depth electrodes at 1.5T with a wbSAR of 2.5 W/kg.^29^ However, one crucial difference between these and the Dixi depth electrodes, likely contributing to the increased heating observed in our study, is the lead length (38 vs. 100 cm).^29^ RF heating is known to increase near resonance conditions, which depend on physical parameters such as lead length, body size, and the electrical properties of the surrounding tissue. Resonance may occur at critical lead lengths, leading to excessive heating even at standard SAR levels. While the specific resonant length varies with the implant and its environment, wires which are partially immersed in tissue—such as SEEG electrodes—may reach resonance at different lengths than fully immersed ones. It is therefore likely that the longer lead length of the Dixi electrodes brought the configuration closer to a resonance condition (see Appendix 1 for further details about the calculation of resonant wire lengths in MRI). This behavior has been demonstrated previously in depth electrodes with varying wire lengths,^30^ and may also be influenced by other factors, including phantom size.^31^ Other relevant factors affecting RF heating include whether the electrode is connected to an amplifier,^23^ the diameter of the wire,^32^ the diameter of the electrode contacts,^27^ the length of the external lead,^22^ and the immersion depth.^31^, ^33^ Given the variety of factors that can influence resonance and RF heating, it is advisable to configure MRI protocols conservatively. Based on our findings, a SAR limit of 0.1 W/kg or less appears appropriate to reduce the heating risk at 1.5 T, with a horizontal bore MRI scanner.

As far as we are aware, only one previous study has examined RF heating of Dixi electrodes,^23^ testing various electrode configurations during MRI in both a phantom and an animal model. That study reported a maximum temperature rise of 4.9°C when the electrodes were disconnected from the amplifier, with temperature increases ranging from 0 to 1.3°C for all other configurations. However, SAR values were not consistently reported across sequences, limiting direct comparisons with the present study. In addition, several differences in experimental setup and testing conditions between the present study and the study by Ciumas et al.,^23^ may account for the differing results. In our study, we deliberately tested worst‐case conditions using the maximum wbSAR and placed the electrode in the thoracic section of the ASTM phantom. This placement was chosen because RF heating is now known to be underestimated in the head portion of the phantom, due to its simplified geometry, which does not accurately reflect anatomical features influencing the effective electrical length of the implant.^34^ The current ASTM 2182 standard therefore recommends measuring RF heating in the worst‐case location of the phantom, in alignment with the measurement of local SAR. For an accurate safety assessment, the results should be rescaled to the highest local SAR at the implant site. Additional differences include the use of a 5‐contact depth electrode in the previous study compared to a 10‐contact Dixi electrode in our study. It also remains unclear how the electric field distribution in the rabbit model used by Ciumas et al.^23^ compares to that in the human body. Most notably, their measurements were limited to the tip contact, which in our experiments consistently showed only minimal heating of 1–2°C, even in configurations where proximal contacts reached temperature increases of up to 48°C.

The stability of the measured temperature changes is underscored by the similar heating measured in the straight‐line configuration across test sessions, particularly in configurations A and B, after repositioning of the electrode and the temperature probes. This straight‐line configuration represents the most common clinical scenario, since the electrode leads are typically left approximately straight, or gently curved and secured under a head dressing for protection and infection control. Tight coiling of the electrode is typically avoided because the electrodes are fragile, and a risk of mechanical damage to the lead must be weighed against the potential benefits when evaluating the risks and benefits of postoperative MRI versus CT.

While this study aimed to assess the worst‐case heating near the left edge of the phantom, repositioning the electrode to a more clinically relevant midline location still resulted in unacceptably high heating (Δ*T* = 29°C). When more than 12 cm of the electrode lead was within the RF coil, the only changes that effectively reduced the heating to 1°C were reducing the SAR to 0.16 W/kg and coiling the electrode with a narrow diameter loop of 6 cm. Coiling the end of the electrode lead into a tight loop has also been recommended for other conductive implants^18^ and is thought to reduce heating by acting as an RF choke and limiting the electric field variation across the loop. At the same time, it also reduces the effective length of the lead wire. Similar effects have been observed when measuring heating as a function of lead length in pacemaker wires, where coiling the wire with a 2.5 cm loop diameter was able to reproduce the heating effects observed when cutting the wire to the same effective lead length.^35^ However, there is also a risk of mechanical damage to the lead when forming tight coils, and such coiling may not be feasible in all clinical scenarios.

## 5 LIMITATIONS

Using a conservative wBSAR limit (e.g., 0.1 W/kg) also helped reduce heating, and is consistent with the limits applied to other intracranial electrodes known to exhibit significant heating at higher SAR levels.^16^, ^17^ However, even at this lower SAR, safe scanning cannot be guaranteed in all scenarios. In addition, the conditions tested in the present study may underestimate the heating when multiple electrodes are positioned in close proximity to each other. The implantation of multiple depth electrodes is a common clinical scenario, but the RF heating in this scenario was not tested in the present study. The results also cannot be extended to scanners with different field strengths or magnetic fields at different orientations relative to the long axis of the patient.

Finally, it is important to note that phantom studies represent a worst‐case approximation and do not account for physiological heat dissipation through tissue perfusion. However, perfusion typically reduces temperature over a longer timescale (minutes),^36^ and is unlikely to significantly offset the rapid heating increases observed in our study.

## 6 CONCLUSION

Dixi microdeep electrodes are susceptible to significant RF‐induced heating during MRI at 1.5 T. Coiling of the external lead to a diameter of 6 cm or less, and using a conservative SAR limit of 0.1 W/kg may substantially reduce the heating risk. However, these results cannot be extended to scanners with different field strengths or magnetic fields at different orientations relative to the long axis of the patient. MRI scanning with these electrodes in situ should be approached with caution, guided by in vitro safety data and awareness of worst‐case heating risks.

## AUTHOR CONTRIBUTIONS

ROT, RL, RK, NK, and GR: conceptualization. RL: methodology. ROT, RL, and RH: investigation. ROT: formal analysis. SR, NK: resources. ROT: writing—original draft. RL, RH, RK, NK, SR, and GR: writing—review and editing.

## CONFLICT OF INTEREST STATEMENT

None of the authors has any conflict of interest to disclose. We confirm that we have read the journal’s position on issues involved in ethics publication and affirm that this report is consistent with those guidelines.

## SOCIAL MEDIA DESCRIPTION

SEEG depth electrodes can heat dangerously during MRI, and different electrode contacts can show large differences in heating.

## Supporting information


**Figure S1:** High‐resolution photos of the electrode (left panels), with the fluoroptic
**Figure S2:** Vertical placement of the lead: the electrode insertion length was 10 cm.

## Data Availability

The data supporting this study’s findings are available from the corresponding author upon request.

## APPENDIX 1 CALCULATION OF RESONANCE LENGTH FOR PARTIALLY IMMERSED WIRES

The wavelength of an electromagnetic wave in free space is given by:

$$\lambda_{0}=\frac{c}{f}=\frac{2\mathrm{\pi c}}{\omega }$$

where *c* is the speed of light (3 × 10^8^ m/s), f is the linear RF frequency (64 MHz for 1.5T), and *ω* is the angular frequency, where *ω* = 2*πf*.

In a nonconducting dielectric medium, the wavelength is reduced by the square root of the dielectric constant of the medium:

$$\lambda =\frac{\lambda_{0}}{\sqrt{\epsilon_{T}}}=\frac{2\mathrm{\pi c}}{\omega \sqrt{\epsilon_{T}}}$$

where $\epsilon_{T}$ is the relative electrical permittivity (dielectric constant) of the surrounding media (1.0 for air and 74.5 for gray matter^27^, ^37^). According to this equation, the resonant half wavelength at 1.5T is 2.43 m in air and 27 cm in tissue. However, for lossy dielectric media with nonzero electrical conductivity, the wavelength is given by:

$$\lambda =\frac{2\mathrm{\pi c}}{\omega \sqrt{\epsilon_{T}}}{\left[\frac{1}{2}+\frac{1}{2}\sqrt{1+{\left(\frac{\sigma }{\omega \epsilon }\right)}^{2}}\right]}^{-\frac{1}{2}}$$

$$\lambda =\frac{2\mathrm{\pi c}}{\omega \sqrt{\frac{\epsilon_{T}}{2}\left(1+\sqrt{1+{\left(\frac{\sigma }{\omega \epsilon }\right)}^{2}}\right)}}$$

where *σ* is the conductivity (0.511 S/m in gray matter) and ϵ is the absolute permittivity, given by $\epsilon_{T}*\epsilon_{0}$, where $\epsilon_{0}$ is the permittivity of free space (8.854 × 10^−12^ F/m).

For cerebral gray matter, the product of ω*ϵ is 4.02 × 10^8^ * 6.6 × 10^−10^ = 0.265 at 64 MHz (1.5T). The factor $\frac{\sigma }{\omega \epsilon }$ is therefore 1.92, and the wavelength is reduced by a factor of 10.85 relative to the wavelength in air, resulting in a resonant wavelength of 43.2 cm and a resonant half wavelength of 21.8 cm.^38^, ^39^

For the ASTM phantom, σ = 0.47 S/m, *ϵ*_T_ = 78, and the product of ω*ϵ is 4.02 × 10^8^ * 6.9 × 10^−10^ = 0.278 at 64 MHz. The factor $\frac{\sigma }{\omega \epsilon }$ is therefore 1.69, and the wavelength is reduced by a factor of 10.75 relative to the wavelength in air, resulting in a resonant wavelength of 43.6 cm and a resonant half wavelength of 21.8 cm.^38^, ^39^

For partially immersed wires, the resonant length has been reported to shift toward odd multiples of the quarter wavelength.^31^
