# Supplementary material for: Safety of postimplantation MRI with Dixi microdeep electrodes in situ: An in vitro evaluation of MRI‐related heating at 1.5T
Source: Epilepsia Open. 2026 Feb 20;11(2):553–63. doi: 10.1002/epi4.70238 (PMC13051839; doi:10.1002/epi4.70238)
Supplement: Supplementary file 1 — Figure S1: High‐resolution photos of the electrode (left panels), with the fluoroptic Figure S2: Vertical placement of the lead: the electrode insertion length was 10 cm. [file EPI4-11-553-s001.pdf]

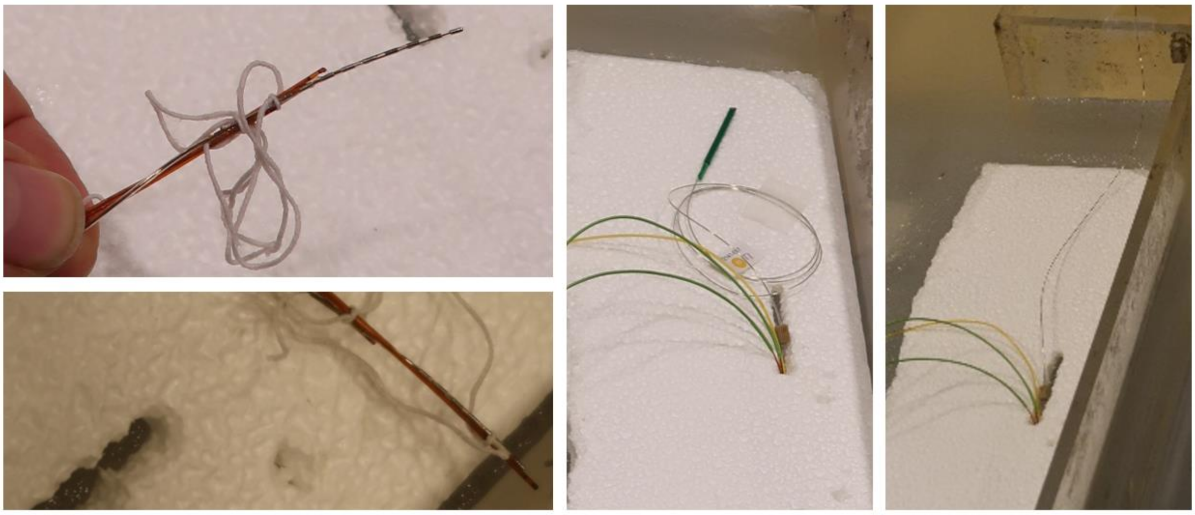

Supplementary Figure 1: High-resolution photos of the electrode (left panels), with the fluoroptic thermometry probes attached to the selected contacts with string. During the measurements the electrode was inserted into the gel through a slit in a thin sheet of Styrofoam positioned on top of the gel at the edge of the tank (middle and right panels). The green and yellow wires are the cables for the thermometry probes, which were inserted into the gel together with the electrode. The free end of the electrode was either coiled in air (middle panel) or positioned in a straight orientation along the z axis in air (right panel).

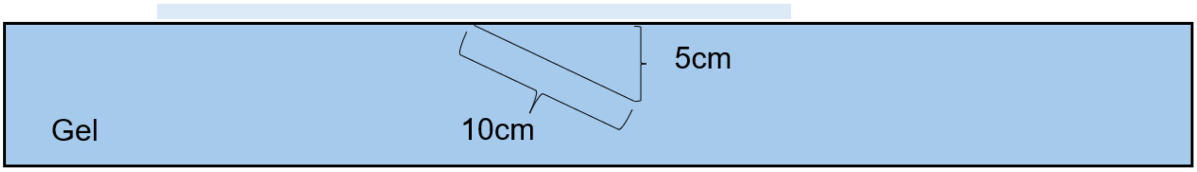

Supplementary Figure 2: Vertical placement of the lead: the electrode insertion length was 10 cm, at an angle of 30 degrees, such that the electrode tip was 5 cm below the surface of the gel. (The depth of gel in the tank is 9 cm, and the electrode reached the grid 4 cm above the base of the tank. The length of the torso section of the tank is 65 cm.)
